# Isolation of Novel Adenovirus from Fruit Bat (*Pteropus dasymallus yayeyamae*)

**DOI:** 10.3201/eid1402.070932

**Published:** 2008-02

**Authors:** Ken Maeda, Eiichi Hondo, Junpei Terakawa, Yasuo Kiso, Numekazu Nakaichi, Daiji Endoh, Kouji Sakai, Shigeru Morikawa, Tetsuya Mizutani

**Affiliations:** * Yamaguchi University, Yamaguchi, Japan; † Rakuno Gakuen University, Ebetsu, Japan; ‡ National Institute of Infectious Diseases, Tokyo, Japan

**Keywords:** Adenovirus, fruit bat, pteropus, letter

## Abstract

Isolation of Novel Adenovirus from Fruit Bat

**To the Editor**: Bats are thought to be one of the most important reservoirs for viruses such as Nipah virus, severe acute respiratory syndrome (SARS) coronavirus, and Ebola virus (*1*). These pathogens became known after extensive surveys of bats following outbreaks. As a first step in investigating unidentified pathogens in bats and to help forecast the potential threat of emerging infectious diseases, we tried to isolate and characterize viruses that persistently infect bats. In the process, we isolated a novel adenovirus from a fruit bat in Japan.

*Pteropus dasymallus yayeyamae*, or Ryukyu flying fox, is a fruit bat of Japan*.* With the permission of the governor of Okinawa, we caught 1 adult male bat of this species and used its spleen and kidneys to establish primary cell cultures. On the 4th passage of the primary adherent cells derived from the spleen, a cytopathic effect (CPE) appeared without any visible microbe, indicating that the cell culture contained a virus. The virus, tentatively named Ryukyu virus 1 (RV1), caused apparent CPE on primary kidney cells derived from a Ryukyu flying fox and on our established bat kidney T1 (BKT1) cells, which were derived from the kidney of a horseshoe bat (*Rhinolophus ferrumequinum*) and transformed with expression plasmid DNA encoding the large T antigen of replication origin-defective simian virus 40.

To identify the virus, RV1, we applied the rapid determination of viral RNA (RDV) system version 1.0 (*2*). However, no viral nucleic acid sequence was detected from an RNA sample in the RV1-infected BKT1 cells. For detection of viral DNA, we developed a system for rapid determination of viral DNA sequences (RDV-D) by minor modification to the RDV system for RNA viruses (*2**–**4*). The results indicated that 2 of the fragments were homologous to the gene encoding the precursor of terminal protein (pTP) of adenoviruses. Further RDV-D analysis showed that 6 fragments (139 bp, DDBJ/EMBL/GenBank accession no. AB302970) were homologous to the pTP gene and that another 6 fragments (316bp, DDBJ/EMBL/GenBank accession no. AB302971) were homologous to the gene encoding the precursor of protein VI (pVI) of adenoviruses. These results indicated that RV1 must belong to the family *Adenoviridae*.

To further confirm that RV1 isolate was an adenovirus, we used PCR and sequencing. We performed the first reaction with the outer primer pair (polFouter and polRouter) of a nested PCR method, targeting the viral DNA polymerase gene with highly degenerate consensus primers that have been described recently (*5*). A fragment of ≈550 bp was amplified from RV1 as well as from human adenoviruses-1, -3, -4, and -7 (data not shown). Sequence analysis of the amplified product (DDBJ/EMBL/GenBank accession no. AB303301) showed that RV1 was homologous to tree shrew adenovirus 1 (70.0% amino acid sequence identity), porcine adenovirus 5 (69.2%), canine adenovirus 1 (68.9%), human adenoviruses-3, -16, -21 and -50 (68.9%), and other viruses (>64.8%) in genus *Mastadenovirus*, but less homologous (46.7%–57.8%) to viruses in other genuses, *Siadenovirus, Aviadenovirus,* and *Atadenovirus*. In addition, a phylogenic tree based on amino acid sequences indicated that RV1 belongs to family *Adenoviridae*, genus *Mastadenovirus* (Figure).

**Figure:**
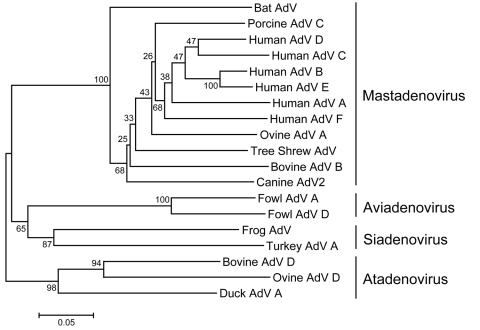
Phylogeny of adenoviruses based on analysis of partial amino acid sequences of DNA polymerase protein. Trees were estimated by using the neighbor-joining method based on the amino acid pairwise distance and MEGA 4.0 software (www.megasoftware.net). Numbers represent percentage bootstrap support (100 replicates).

Electron microscopy of RV1-infected BKT1 cells indicated that RV1 accumulated in the nucleus and that the size of capsids was 60–70 nm (data not shown). Restriction endonuclease analysis of the RV1 genome indicated that the genome was ≈20–30 kbp (data not shown). These features are consistent with RV1 being an adenovirus.

Until now, a number of RNA viruses have been isolated from bats, but isolation of DNA virus is rare (*1*). The isolation of the novel adenovirus seems to be possible because of usage of the primary cells originated from the host; DNA viruses might have more restricted host range than RNA viruses and require host-originated cells for the growth. In addition, our success in DNA virus isolation might have resulted from usage of the adult animal latently and persistently infected with DNA viruses such as adenovirus and herpesvirus.

In conclusion, we isolated a novel virus from a fruit bat. This virus was isolated from a healthy bat, which suggests that the virus may persistently infect fruit bats. Although its pathogenicity for humans is still unknown, knowledge of RV1 will be useful in epidemiologic studies of infectious diseases emerging from bats because persistently infecting viruses might be isolated together with primary pathogens. We are planning to establish cell lines from bats and isolate more viruses from persistently infected bats.
