# Isolation of Novel Adenovirus from Fruit Bat (*Pteropus dasymallus yayeyamae*)

**DOI:** 10.3201/eid0805.020503

**Published:** 2002-05

## Correction, Vol. 8, No. 4

In “Antimicrobial Use and Antimicrobial Resistance: A Population Perspective,” by M. Lipsitch and M.H. Samore, the following references appeared out of order: nos. 10, 14, and 19. The corrected version appears online at http://www.cdc.gov/ncidod/EID/vol8no4/01-0312.htm . We regret any confusion this error may have caused.

